# Jacob Heine (1800–1879)

**DOI:** 10.1007/s00415-017-8454-7

**Published:** 2017-03-16

**Authors:** Krzysztof Pietrzak, Andrzej Grzybowski, Jacek Kaczmarczyk

**Affiliations:** 1 Department of Orthopaedics and Traumatology, University of Medical Sciences, Poznań, Poland; 2 Department of Ophthalmology, Poznań City Hospital, Poznań, Poland; 3 Department of Ophthalmology, University of Warmia and Mazury, Olsztyn, Poland

Jacob Heine was born on 16 April 1800 in Lauterbach in Germany. His father, Martin Heine, was a smith and a farmer, who on the farm used the equipment that he himself improved or even invented. He also constructed a special bed for rehabilitation exercises. Since early childhood Jacob had keenly been interested in science and technological novelties. First, he studied Latin and theology, but he followed the advice of his uncle, Johann Georg Heine (1771–1838), owner of an orthopaedic establishment in Würzburg, and started medical studies at Würzburg University. While studying, he and his cousin Bernhard Heine (1800–s1846), who was later to become the first surgeon to perform an osteotomy to straighten bones, also received practical training at their uncle’s institute. In 1827 Jacob Heine presented a doctoral thesis on the occlusion of the subclavian artery [1]. During his studies and for a year and a half after his graduation, Jacob was an assistant to Johann Lukas Schönlein (1793–1864). At the same time, he was also involved in his uncle’s orthopaedic institute. In 1829, Johann Georg moved to The Netherlands and left the institute in charge of Jacob and Bernhard.

In the same year, with the support of the Baden-Württemberg authorities, Jacob Heine became head of the orthopaedic hospital at Bad Cannstatt, and kept the position until he retired in 1865. He set up an orthopaedic and rehabilitation establishment, with a number of pools and exercise equipment. His insightful observations of patients, which he continued for many years, resulted in a monograph describing 29 cases [2]. There had been short reports on the poliomyelitis before [3, 4], yet Heine did not mention them in his monograph. In his book, Heine made a clear distinction between his cases and patients with cerebral palsy, mental retardation, rickets, or encephalitis. Heine’s patients had flaccid paralysis, affecting one or two limbs, or hemiparesis. The author associated their condition with an early childhood infection with general symptoms, often present during teething. Heine’s monograph included a description of the observations, which lasted for 14 years, as well as recommended treatment: from baths and massages to contracture-preventing exercises. Heine described residual deformities of limbs and a full set of reparative orthopaedic treatments, including the cutting of the tendon. Drawing on family experience, Heine also developed orthopaedic appliances, such as splints and suspenders, which helped patients with flaccid paralysis to move.

Heine attributed the condition to an inflammation of the anterior horns of the spinal cord. The same idea was put forward by John Abercombie (1780–1844), who described similar disorders [5], though his name was not mentioned by Heine. What is important is that Heine described a disease that in his times, before the 1887 epidemic spread across the northern hemisphere, which was a very rare occurrence. In his next monograph, Heine described as many as 120 patients [6]. His descriptions of the disease and its treatment had not changed for many years even though there were many attempts to modify the therapy.

Heine’s first monograph met both with lively interest and considerable controversy. The dispute focused on whether the inflammation of anterior horns was, indeed, the cause of the disease. Heine supported his theory with results of experiments on animals and clinical pictures of other diseases with accompanying damage to the anterior horns of the spinal cord. Heine’s theory came in for much criticism. The few anatomopathological studies of flaccid paralysis, which Heine described, were not sufficient to provide an unambiguous confirmation. The theory was finally confirmed in 1870 by Jean-Martin Charcot (1825–1893) and his associates [7].

In 1890, when the disease spread across Europe and the United States, Karl Oskar Medin (1847–1914), a Swedish pediatrician, described the disease again [8], pointing to its epidemic character.

The name ‘Heine-Medin disease’ was first officially used in 1907 [9], thanks to Otto Ivar Wickman (1872–1914), a Swedish doctor and Karl Medin’s student. The name seems to be a fair reflection of the contributions of the individual scientists. Heine not only described the disease in detail, carried out observations of its effects, often lasting for many years, and provided a systematic description of the symptoms, but he also proposed a treatment plan, including reparative surgery and appliances for supporting patients. His treatment method, as possibly effective, lasted for decades. If Heine had mentioned the authors of the earlier short reports on the disease, this would have made his monograph even more worthy.

Heine’s accomplishments brought him fame also outside the world of medicine. As a honorary member of numerous national and international medical associations, he was also awarded medals by the rulers of Baden-Württemberg and the Russian Empire.

Since 1831, Heine had been married to Henriette Ludovike Camerer (1807–1874). The marriage was harmonious and Heine’s wife was a great support. She taught young patients at Heine’s institute. They had seven children, one of whom, Carl Wilhelm Heine (1838–1877), became an outstanding surgeon. Heine ran the orthopaedic institute until his retirement in 1865. He died on 12 November 1879 in Cannstatt.
